# Proteasome Subunits Regulate Reproduction in *Nilaparvata lugens* and the Transovarial Transmission of Its Yeast-like Symbionts

**DOI:** 10.3390/insects16090895

**Published:** 2025-08-27

**Authors:** Xin Lv, Jia-Yu Tu, Qian Liu, Zhi-Qiang Wu, Chen Lin, Tao Zhou, Xiao-Ping Yu, Yi-Peng Xu

**Affiliations:** 1 Zhejiang Provincial Key Laboratory of Biometrology and Inspection & Quarantine, China Jiliang University, Hangzhou 310018, China; lx2481476963@163.com (X.L.); i1105684544@163.com (J.-Y.T.); 17344135037@163.com (Q.L.); wuzq1101@163.com (Z.-Q.W.); l1657088721@163.com (C.L.); zhout910@gmail.com (T.Z.); yxp@cjlu.edu.cn (X.-P.Y.); 2 Key Laboratory of Microbiological Metrology, Measurement & Bio-Product Quality Security, State Administration for Market Regulation, Hangzhou 310018, China

**Keywords:** *Nilaparvata lugens*, proteasome, RNAi, reproduction, yeast-like symbionts, transovarial transmission

## Abstract

The brown planthopper, *Nilaparvata lugens*, a major rice pest, harbors yeast-like symbionts (YLSs) that form mutualistic relationships with the host, significantly influencing its development and reproduction. As proteasome subunits play major roles in the assembly and functional maintenance of the proteasome, but their regulation on the YLSs in *N. lugens* are unclear. In this study, we analyzed the spatiotemporal and temporal expression patterns of five *N. lugens* proteasome subunits (*NlPSMA2*, *NlPSMB5*, *NlPSMC4*, *NlPSMD10*, *NlPSMD13*), and further verified their functions on the transovarial transmission of YLSs, in addition to the reproduction of *N. lugens*, based on RNA interference (RNAi). The results showed that *NlPSMA2*, *NlPSMB5*, *NlPSMC4*, *NlPSMD10*, and *NlPSMD13* were highly expressed in ovarian follicular cells of *N. lugens* upon sexual maturation. After suppressing the expression of these genes by RNAi, *N. lugens* exhibited a shortened lifespan, abnormal pear-shaped follicles, and impaired oviposition capacity, but the number of YLSs in the whole body and the oocyte of *N. lugens* were significantly increased. These results indicate that the proteasome subunits play crucial roles in the reproduction of *N. lugens* and the transovarial transmission of its YLSs.

## Simple Summary

The brown planthopper *Nilaparvata lugens* (Stål) (Hemiptera: Delphacidae) is one of the major pests in rice. Yeast-like symbionts (YLSs) are primary endosymbionts of *N. lugens*, and significantly influence the growth, development, and reproduction of *N. lugens*. This study investigated the role of proteasome in *N. lugens*. Our results demonstrated that all five proteasome subunits (*NlPSMA2*, *NlPSMB5*, *NlPSMC4*, *NlPSMD10*, *NlPSMD13*) played important roles in the reproduction of *N. lugens*, and proteasome regulated the transovarial transmission of YLSs. Given the functional importance of the proteasome subunits, they represent potential targets for controlling *N. lugens*.

## 1. Introduction

The brown planthopper *Nilaparvata lugens* (Stål) (Hemiptera: Delphacidae) is a serious pest of rice causing significant economic losses in Asian rice cultivation areas [1,2,3]. It feeds on the sap of the rice phloem, leading to plant wilting, increase in the rate of empty grain, and a sudden decrease in yield [4,5,6]. The current means for controlling *N. lugens* mainly rely on chemical pesticides, but chemical control leaves drug residues and causes environmental pollution and other problems. Additionally, *N. lugens* has developed resistance to a variety of insecticides [7,8,9], and it is therefore urgent to find new, green, safe, and efficient control strategies. Recent studies have shown that yeast-like symbionts (YLSs) in brown planthopper play key roles in host nutrient metabolism, development, reproduction, and immune defense [10,11]. These YLSs are transmitted from generation to generation through transovarial transmission, providing important help for host adaptation to the environment and enhancing survival rates [12]. Therefore, controlling the vertical transmission of YLSs is expected to be a new strategy for controlling brown planthopper.

It has been found that *N. lugens* oocyte allows the invasion of YLSs only during the late vitellogenesis stage. During vitellogenesis, the morphological differentiation of follicular cells surrounding oocyte is closely related to the polar deposition of vitellogenin (Vg) and lipids in oocyte, which indirectly regulates the invasion process of YLSs [13]. In this case, the epithelial plug structure formed by the follicular cells that are located posterior to the oocyte is the sole channel for YLSs to enter the oocyte [13]. Our previous experiment found that during the invasion of YLSs into the epithelial plug of *Nilaparvata lugens* ovarioles, the expression levels of five proteasome subunit genes (*PSMA2*, *PSMB5*, *PSMC4*, *PSMD10*, and *PSMD13*) were specifically upregulated in the epithelial plug. Our findings suggest that these proteasome subunits may regulate protein degradation or catalytic activity, and could then facilitate the transovarial transmission of YLSs.

Proteasome subunits are able to control proteasome assembly and substrate recognition, as well as dynamically regulate protein homeostasis, and play pivotal roles in cell cycle, apoptosis, metabolism and development, and their aberrant is directly associated with tumor progression, reproductive disorders, and embryonic lethality [14]. Proteasome plays important roles in oocyte meiosis resumption, spindle assembly, polar body emission, and pronuclear formation [15]. Previous studies have found that the ubiquitin–proteasome system sequentially degrades maternal proteins, which is necessary for the normal initiation of ZGA (zygotic genome activation) and the normal progression of MZT (maternal-to-zygotic transition) in early mouse embryos [16]. Proteasome 26S regulatory subunit 6B (PSMC4) deficiency triggered an embryonic lethal phenotype in a mouse model [17]. The silencing of *proteasome 20S subunit-A6* (*Prosα6*) culminated in the impairment of oocyte maturation at the early stages of oogenesis in *Rhodnius prolixus* [18]. However, there are only a few studies on the function of proteasome in insects. Knockdown of proteasome 26S non-ATPase regulatory subunits and regulatory subunits in *N. lugens* has been found to significantly reduce the protein hydrolyzing activity of the proteasome, impairing ovary development and oocyte maturation [19,20], but their functions related to YLSs and the function of other proteasome subunits in *N. lugens* are still unknown.

In this study, we focused on the five proteasome subunits (*PSMA2*, *PSMB5*, *PSMC4*, *PSMD10*, and *PSMD13*) that were specifically upregulated in the epithelial plug of ovarioles. We analyzed their spatiotemporal expression patterns and investigated their functional roles in *N. lugens* using RNAi.

## 2. Materials and Methods

### 2.1. Insect Rearing

The *N. lugens* population (source: Yuyao, Zhejiang, China) was maintained in an intelligent artificial climate chamber with the following environment: 27 °C ± 1 °C, 60% humidity, and photoperiod L:D = 16 h:8 h. The *N. lugens* population was reared on rice variety “Taichung Native 1”.

### 2.2. cDNA Synthesis

Total RNA was extracted from adult female *N. lugens* samples using the MiniBEST Universal RNA Extraction Kit (Takara Bio, Dalian, China). The cDNA was obtained by reverse transcription of the synthesized RNA using the PrimeScript^TM^ II 1^st^ Strand cDNA Synthesis Kit (Takara Bio), with random primer (N7) and Oligo (dT).

### 2.3. RNA Interference

Primers for the DNA template required for dsRNA synthesis were designed, with T7 polymerase promoter sequence added to the 5′ end (Table 1). After the DNA template was amplified and purified, dsRNA was then synthesized using the MEGAscript^TM^ T7 Transcription kit (Ambion, Austin, TX, USA). The quality of this dsRNA product was verified by 1% agarose gel electrophoresis and NanoDrop 2000 spectrophotometer (Thermo Fisher Scientific, Waltham, MA, USA) and stored for backup. *GFP* dsRNA (ds*GFP*) was used for a negative control as previously described [21].

Adult females within 12 h or 24 h of emergence were selected for subsequent experiments. Approximately 50 nL of dsRNA (2000 ng/μL) was injected into the abdomen of each newly emerged brachypterous adult female using a manual microsyringe for RNA interference.

### 2.4. Real-Time Quantitative PCR Analysis

In this study, we employed real-time quantitative PCR (qPCR) to analyze the proteasome subunit gene mRNA levels in different *N. lugens* samples. The qPCR primer pairs for amplification of the proteasome subunit genes were designed (Table 2), and qPCR was performed on Step One Plus (ABI, Foster City, CA, USA) using TB Green^®^ Primix Ex Taq^TM^ II (Tli RNaseH Plus) (Takara Bio). qPCR procedure was as follows—94 °C, 30 s; 94 °C, 5 s, 60 °C, 30 s, and 40 cycles—and the corresponding data were collected at the end of the reaction. The relative expression level of target genes was calculated by the 2^−ΔΔCt^ method, taking *N. lugens 18S rDNA* as internal reference [21].

To analyze the developmental expression patterns of the proteasome subunit genes, total RNA was extracted from first–fifth nymphs, 1-, 3-, 5-, and 7-day-old brachypterous adult females, and 1- and 2-day-old brachypterous adult males. To analyze their tissue-specific expression pattern, total RNA was extracted from the head, thorax, gut, ovaries, and fat bodies of brachypterous adult females. To analyze their expression change after dsRNA injection, total RNA was extracted from *N. lugens* on the 2nd, 3rd, and 5th day post-dsRNA injection.

To analyze the relative copy number of YLSs’ genomes after dsRNA injection, genomic DNA was extracted from *N. lugens* on the 3rd day post-dsRNA injection. The number of YLSs in whole insects after RNAi was explored. In each replicate experiment of different treatment, five females of *N. lugens* were taken to extract genomic DNA using the Animal Genomic DNA Rapid Extraction Kit (Sangon Biotech, Shanghai, China) on the 3rd day post-dsRNA injection. The relative copy number of the genomes of the YLS *Entomomyces delphacidicola* and *Moesziomyces* sp. was examined by qPCR with design primer pairs (*Entomomyces delphacidicola*-qF/*Entomomyces delphacidicola*-qR; *Moesziomyces* sp.-qF/*Moesziomyces* sp.-qR) (Table 2) using the same procedure mentioned above. The *N. lugens 18S rDNA* was also used as the internal reference.

### 2.5. Dissection Observations and Fertility Analysis

Five *N. lugens* females were randomly collected from the experimental and control groups and dissected at intervals of 24 h after RNA interference. Inverted microscope was used to observe the morphology of ovaries, intestines and fat bodies of *N. lugens*. At the same time, three replicates of 30 adult females were set up in the experimental and control groups to observe the effects of RNA interference on the growth, development, and survival rate of *N. lugens*.

In the counting of YLSs in oocytes, any 10 dsRNA-treated brachypterous adult females of *N. lugens* were dissected, and 3~4 mature oocytes were taken from each individual. The oocytes were then treated with a 35% sodium hypochlorite solution in order to disintegrate the oocytes and cause them to release the YLSs. After the YLSs were all released from the oocytes and completely dispersed, they were photographed using NIS-Elements D 3.10 (Build 578) (Nikon, Tokyo, Japan) and the number of YLSs was counted.

In the observation of fat bodies, *N. lugens* were dissected on the 3rd day post-dsRNA injection, and fat bodies within hemolymph were dispersed in 30 μL PBS solution.

### 2.6. Immunofluorescence

The expression of PSMC4 protein in the ovaries of *N. lugens* adult females was observed by immunofluorescence analysis. The anti-PSMC4 antibody used was an anti-human mouse monoclonal antibody (Santa Cruz, Dallas, TX, USA), whose antigenic sequence has 91.62% similarity to *N. lugens* PSMC4. Total proteins of *N. lugens* and its ovaries were extracted, and the specificity of the PSMC4 antibody was verified using Western blot. After DAB color development, the results showed the appearance of a single specific brown target band, which was in accordance with the predicted size of the PSMC4 protein (Figure S1), suggesting that the purchased PSMC4 antibody has strong specificity for the endogenous PSMC4 protein of *N. lugens*.

For immunofluorescence, the PSMC4 antibody was selected for the primary antibody at a dilution of 1:100. Goat anti-mouse IgG antibody conjugated with DyLight 594 fluorescent dye (Abbkine, Santa Ana, CA, USA) was used as the secondary antibody at a dilution of 1:500. DAPI dye (Abbkine, Santa Ana, CA, USA) was diluted at 1:500 for the staining of cell nuclei. The ovaries of *N. lugens* were stained and observed with a laser scanning confocal microscope (Leica SP8, Mannheim, Germany).

## 3. Results

### 3.1. Developmental and Tissue-Specific Expression of NlPSMA2, NlPSMB5, NlPSMC4, NlPSMD10, and NlPSMD13

**Figure 1:**
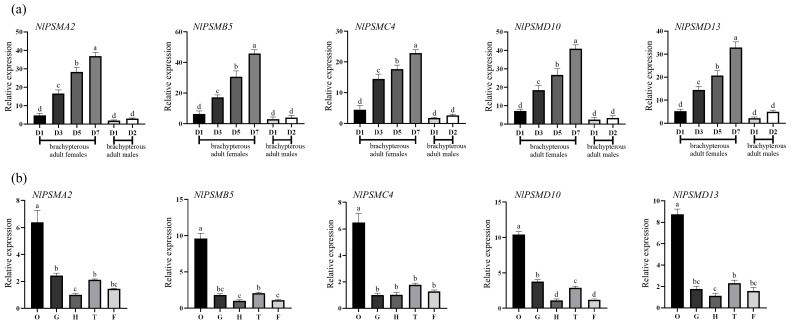
Expression of *NlPSMA2*, *NlPSMB5*, *NlPSMC4*, *NlPSMD10,* and *NlPSMD13* during different developmental stages and in different tissues of *N. lugens*. (**a**) The expression patterns of these five genes during differential developmental stages, including first to fifth nymphs, brachypterous adult females (1, 3, 5, and 7 days old), and brachypterous adult males (1–2 days old) (“D” represents “day-old” adults). (**b**) The expression patterns of these five genes in the head (H), ovaries (O), gut (G), thorax (T), and fat bodies (F) of brachypterous adult females. Different lower-case letters above the bars indicate significant differences at *p* < 0.05 (one-way ANOVA performed using GraphPad Prism Software 8.0).

qPCR results showed that *NlPSMA2*, *NlPSMB5*, *NlPSMC4*, *NlPSMD10,* and *NlPSMD13* were expressed in both females and males, with a large increase in 3-, 5-, and 7-day-old brachypterous adult females (Figure 1a). They were also expressed in all tissue sites, with the highest expression in the ovaries of adult females, approximately six times higher compared to the gut and head (Figure 1b). When three days old, female *N. lugens* begins to reach sexual maturity, and its ovarian development begins. At the same time, a significant vitellogenesis occurs, which relatively coincides with the time when a large number of YLSs enter oocytes. This suggests that *NlPSMA2*, *NlPSMB5*, *NlPSMC4*, *NlPSMD10*, and *NlPSMD13* have a function in female ovarian development and may be associated with the transovarial transmission of YLSs.

### 3.2. Function Validation of NlPSMA2, NlPSMB5, NlPSMC4, NlPSMD10, and NlPSMD13 by RNA Interference

**Figure 2:**
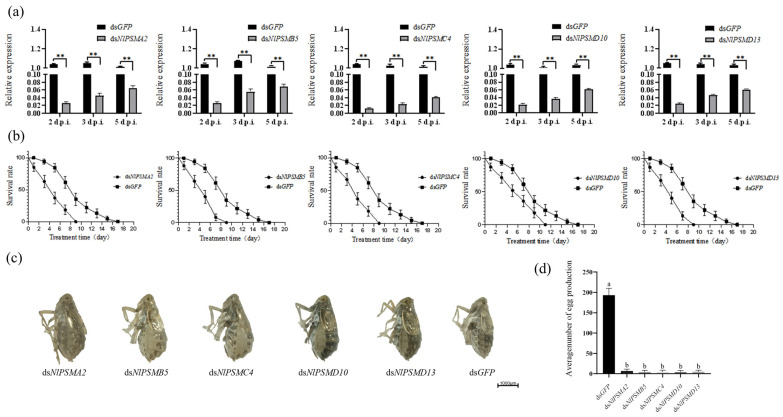
The effects of RNA interference on *N. lugens*. (**a**) Downregulation of *NlPSMC4* expression after the ds*NlPSMC4* injection (d.p.i. represents days post-dsRNA injection). (**b**) Survival rate of *N. lugens* after dsRNA injection. (**c**) Differences in morphology of *N. lugens* on the 4th day after dsRNA injection. (**d**) *N. lugens* egg production after dsRNA injection. Data were analyzed using one-way ANOVA in GraphPad Prism 8.0 software. “**” represents *p* < 0.01 significant difference, and different lower-case letters above the bars indicate *p* < 0.05 significant difference.

The expression of *NlPSMA2*, *NlPSMB5*, *NlPSMC4*, *NlPSMD10,* and *NlPSMD13* genes was significantly decreased (*p* < 0.01) after dsRNA injection (Figure 2a). This indicates that the expression level of these genes can be effectively reduced by RNAi technology.

*N. lugens* injected with ds*GFP* survived for a maximum of 17 days, whereas those injected with ds*NlPSMA2*, ds*NlPSMB5*, ds*NlPSMC4*, ds*NlPSMD10*, or ds*NlPSMD13* survived for 9–11 days (Figure 2b), a significantly difference compared to the ds*GFP*-treated (control) group (*p* < 0.01). After being injected with dsRNA, *N. lugens* adult females had difficulty in oviposition, and their abdomen became swollen (Figure 2c), with a significant difference in average number of eggs produced compared to the control (*p* < 0.01) (Figure 2d) and zero hatching rate.

**Figure 3:**
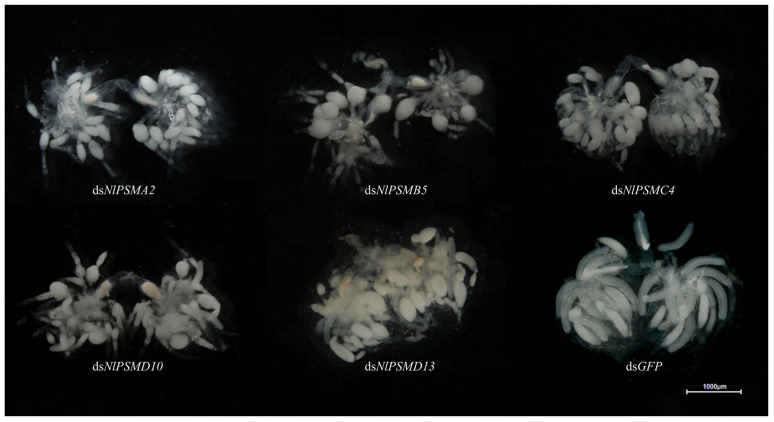
Effect of dsRNA injection on the development of ovaries in *N. lugens* on the 4th day post-dsRNA injection.

After injection, it was found that the ovaries of *N. lugens* in the control group were fully developed, and the typically banana-shaped eggs could be clearly seen, while the ovaries of the adult females in the experimental group injected with ds*NlPSMA2*, ds*NlPSMB5*, ds*NlPSMC4*, ds*NlPSMD10,* or ds*NlPSMD13* developed abnormally, with mostly pear-shaped follicles, indicating that the downregulation of the expression of *NlPSMA2*, *NlPSMB5*, *NlPSMC4*, *NlPSMD10,* or *NlPSMD13* had a great effect on the ovarian development of *N. lugens* (Figure 3).

Since *NlPSMA2*, *NlPSMB5*, *NlPSMC4*, *NlPSMD10*, and *NlPSMD13* exhibited similar phenotypes after RNAi, we selected *NlPSMC4* as the representative for further investigation into its role in the transovarial transmission of YLSs.

**Figure 4:**
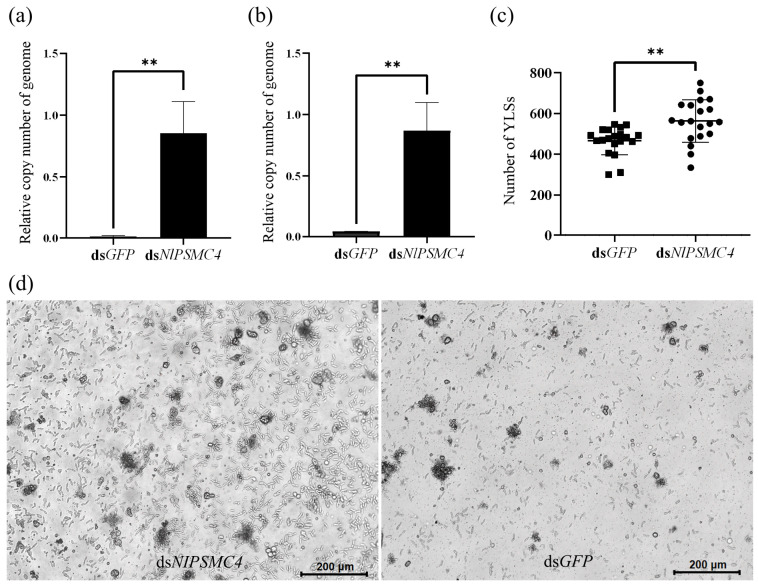
YLSs in *N. lugens*. (**a**) Relative genome copy number of *Entomomyces delphacidicola* in *N. lugens* on the 3rd day post-dsRNA injection. (**b**) Relative genome copy number of *Moesziomyces* sp. in *N. lugens* on the 3rd day post-dsRNA injection. (**c**) Average number of YLSs in the oocytes of *N. lugens* on the 5th day post-dsRNA injection. (**d**) Fat bodies from the female of *N. lugens* on the 5th day post-dsRNA injection. Data were analyzed using one-way ANOVA in GraphPad Prism 8.0 software, and “**” represents *p* < 0.01 significant difference.

We further determined the relative copy number of genomes of YLSs in whole adult females. qPCR results showed that the relative copy number of genome of symbiont *Entomomyces delphacidicola* or *Moesziomyces* sp. was significantly higher in the ds*NlPSMC4* injection group compared with ds*GFP* injection group (Figure 4a,b).

Because the follicles of brachypterous adult females that emerged within 12 h were abnormal and difficult to collect after injection with ds*NlPSMC4*, one day-old brachypterous adult females were chosen for dsRNA injection, and oocytes were collected to count the number of YLSs. It was found that the number of YLSs increased in the oocytes after ds*NlPSMC4* treatment (Figure 4c), indicating that the downregulation of *NlPSMC4* expression can promote the transovarial transmission of YLSs.

Fat body cells of adult females in the experimental group were relatively more dispersed, and the number of fat body cells in small clusters was significantly higher compared to the control group, and more YLSs were also found in the hemolymph. This result indicates that the downregulation of *NlPSMC4* expression has a great impact on the fat bodies and YLSs (Figure 4d).

### 3.3. Immunofluorescence Analysis of PSMC4 Expression

**Figure 5:**
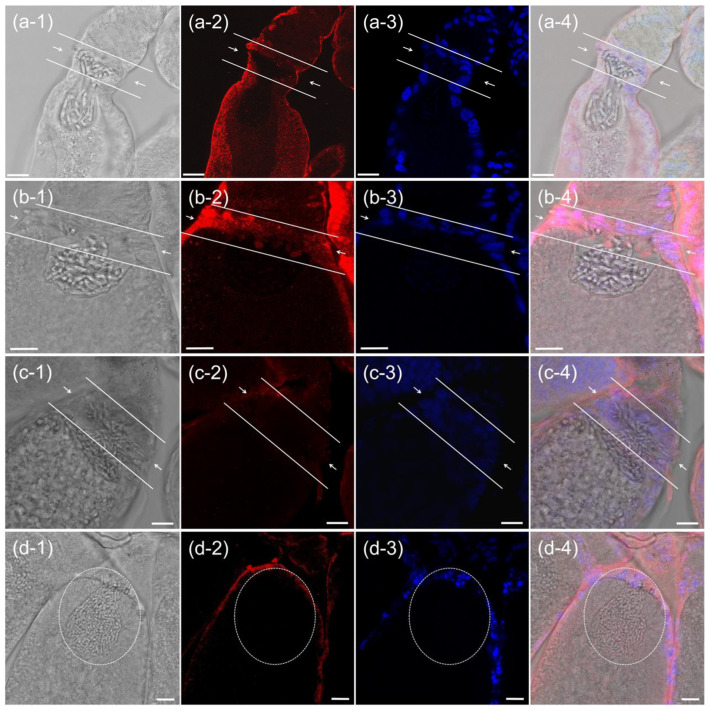
Expression of *Nl*PSMC4 at the epithelial plugs during the entry of YLSs into ovary (**a-1**–**b-4**) in the ds*GFP* treatment group and (**c-1**–**d-4**) in the ds*NlPSMC4* treatment group. The arrow indicates the epithelial plug. Red fluorescent signal represents the expression of *Nl*PSMC4, and blue fluorescent signal represents the nucleus. Scale bar: 25 μm.

Because *N. lugens* proteasome subunit genes were found to be highly expressed in the ovaries of *N. lugens*, immunofluorescence was used to conduct a more detailed observation of its expression in the ovaries, taking *Nl*PSMC4 as the label. The results showed that *Nl*PSMC4 was highly expressed in follicular cells surrounding the oocyte and higher at the epithelial plug of the ovariole. At the epithelial plug, the expression of *Nl*PSMC4 was stable during the entry of YLSs into the epithelial plugs in large numbers and then into a cluster (Figure 5a,b), suggesting that *Nl*PSMC4 plays a continuous role in the entry of YLSs into the oocytes.

After the ds*NlPSMC4* injection, the expression of *Nl*PSMC4 in the follicular cells was weakened and spatially disorganized, and the nuclei of the follicular cells showed varying degrees of degradation, indicating that they were undergoing apoptosis (Figure 5c,d). In addition, there was an abnormal accumulation of YLSs on the side of the epithelial plug (significantly more than the number of YLSs in normal oocytes), and the morphological boundary of the YLS ball was blurred (Figure 5c,d). This result is consistent with the counting result of YLSs in oocytes mentioned above.

## 4. Discussion

Previous studies on the proteasome ATPase subunits (PSMC1~PSMC6) and non-ATPase regulatory subunits (PSMD1~PSMD14) of *N. lugens* have indicated their importance in the reproduction, because knockdown of these genes impaired ovary development and oocyte maturation, leading to egg-laying and hatching failures [19,20]. In the present study, we further confirmed the importance of proteasomes in the reproduction of *N. lugens*, since RNAi of proteasome subunit genes (*NlPSMA2*, *NlPSMB5*, *NlPSMC4*, *NlPSMD10,* and *NlPSMD13*) resulted in abnormal follicles in ovaries, reduced fertility, and shortened lifespan of *N. lugens*. In addition, we found that the expression levels of these proteasome subunit genes were relatively low in the females within one day after emergence, but they sharply increased as the adult females reached sexual maturity. Additionally, proteasomes were abundantly present in the follicular cells surrounding the oocyte and were even more concentrated in the epithelial plug when YLSs entered it. Furthermore, RNAi of proteasome subunit *NlPSMC4* expression remarkably increased the number of YLSs in the abdomen and mature oocytes, indicating the regulation of proteasomes in the transovarial transmission of YLSs.

According to the analysis of Wang et al. (2021) [19] and Cheng et al. (2022) [20], knockdown of proteasome expression decreased the proteolytic activity of the proteasome, downregulated triacylglycerol lipase and Vg transcription, as well as CYP307A2 transcription and 20E synthesis, thereby leading to the defective absorption and utilization of nutrients in developing oocytes. Specifically, we discovered the abnormality of the follicles, whose form changed from the normal banana-shaped to pear-shaped after RNAi, which might be due to the deformation of follicular cells surrounding the oocyte, observed in further immunofluorescence. The functional imbalance of cellular homeostasis weakens the ability of cells to resist physiological and environmental stress [22,23,24]. Proteasome plays a vital role in maintaining cellular homeostasis by degrading misfolded, damaged, or excess proteins [25]. As a part of the 26S proteasome, proteasome subunits play a major role in the assembly and functional maintenance of the proteasome. Knockdown of proteasome subunits has been found to significantly inhibit cell proliferation, cell cycle and migration in vitro and in vivo, and significantly promote apoptosis [26,27]. Therefore, RNAi of proteasome subunit expression could lead to the apoptosis of follicular cells, deforming them and resulting in the transformation of follicles into pear-shaped ones.

Another focus of our study was the situation of YLSs in *N. lugens*. YLSs in the whole body, hemolymph, and oocytes, increased after knockdown of proteasome expression. This result might be explained as follows. The proteasome is an essential component of the innate immune system, participating in the degradation of signaling molecules that regulate immune pathways defending against pathogens like fungi, bacteria, and viruses [28,29]. We speculate that the downregulation of proteasome subunit expression may impair these immune pathways, weakening the overall immune capacity of *N. lugens* and consequently leading to the increase in YLSs in its body. Likewise, the follicular cells at the epithelial plug, which were deformed, cannot prevent more YLSs from entering the oocyte. In addition, RNAi of proteasome expression impaired the function of proteasome in maintaining cellular homeostasis, so the fat body cells were dispersed and unable to control the release of YLSs originally encapsulated within the fat bodies into the hemolymph. It is speculated that the downregulation of proteasome expression impacts the transovarial transmission of YLSs in *N. lugens* by influencing the host’s immunity and the cellular homeostasis.

However, in our previous experiments, knockdown of *N. lugens* death-associated protein-1 (DAP-1) also impaired ovarian development, reduced the number of mature oocytes without inducing abnormal follicles, but decreased YLS abundance in oocytes [10]. These contrasting outcomes suggest that a sophisticated regulatory mechanism in *N. lugens* that can maintain the homeostatic balance of YLS quantity in oocytes.

## 5. Conclusions

In summary, we found that knockdown of proteasome subunits (*NlPSMA2*, *NlPSMB5*, *NlPSMC4*, *NlPSMD10,* and *NlPSMD13*) led to premature death of brown planthopper, deformed follicular development, and blocked ovulation. Additionally, reduced proteasome expression increases the number of YLSs in the whole body and oocytes of *N. luges*, indicating that proteasome plays a crucial role in reproduction and regulates the transovarial transmission of YLSs. This finding may reveal a key regulatory role of the proteasome in the interaction between *N. lugens* and symbionts and provide new ideas for pest control targeting symbionts. Given the functional importance of these proteasome subunits, they represent potential targets for controlling *N. lugens*. Further research could explore whether feeding dsRNA can achieve the same effect as dsRNA injection, and if the results are promising, we could use genetically modified rice that generates dsRNA for agricultural pest control.

## Figures and Tables

**Table 1:** The primers used for synthesizing dsRNA in this study.

Primers	Primer Sequence (5′-3′)
ds*NlPSMA2*-F	GGATCCTAATACGACTCACTATAGGGATCGGCCCTATCTGTTC
ds*NlPSMA2*-R	GGATCCTAATACGACTCACTATAGGGCATCACACACACCCACC
ds*NlPSMB5*-F	GGATCCTAATACGACTCACTATAGGGCATGGGCCTCTCCATGG
ds*NlPSMB5*-R	GGATCCTAATACGACTCACTATAGGCTCGGAAATTTTGATCCA
ds*NlPSMC4*-F	GGATCCTAATACGACTCACTATAGGCAACTAGAGTTTTTGGCT
ds*NlPSMC4*-R	GGATCCTAATACGACTCACTATAGGGCATTGCTGTGTTTGTGC
ds*NlPSMD10*-F	GGATCCTAATACGACTCACTATAGGGCATTTCGAAATCGTGAA
ds*NlPSMD10*-R	GGATCCTAATACGACTCACTATAGGGCATGAGACCTAGTGAGG
ds*NlPSMD13*-F	GGATCCTAATACGACTCACTATAGGGCTCAAATCGAAGAACTC
ds*NlPSMD13*-R	GGATCCTAATACGACTCACTATAGGGAGAACTTTGCATAGTGC
ds*GFP*-F	GGATCCTAATACGACTCACTATAGGGATACGTGCAGGAGAGGAC
ds*GFP*-R	GGATCCTAATACGACTCACTATAGGGCAGATTGTGTGGACAGG

**Table 2:** The primers used for qRT-PCR in this study.

Primers	Primer Sequence (5′-3′)
*NlPSMA2*-qF	CACCGTCCGTAGGAATAAAAGC
*NlPSMA2*-qR	GGACCCATACCACTGTAGACCATT
*NlPSMB5*-qF	GCTTTAGCAGATGTATGTGGAATG
*NlPSMB5*-qR	ACCTGATTTTGACGGGTTTTC
*NlPSMC4*-qF	TGGAACTGCCGCTCACTC
*NlPSMC4*-qR	CCCTCGCCCAGGTATTTT
*NlPSMD10*-qF	AAGCCGTTCCGAAGTAGC
*NlPSMD10*-qR	CAGCCAAATCAAGAGGTGTT
*NlPSMD13*-qF	CTCTTCGCTATCTCGGCTGTA
*NlPSMD13*-qR	GCCACTCATTGGGTGTATTTT
*Entomomyces delphacidicola*-qF	TCCCTCTGTGGAACCCCA
*Entomomyces delphacidicola*-qR	GGCGGTCCTAGAAACCAACA
*Moesziomyces* sp.-qF	TGATGCCCCTTAGATGTTCCG
*Moesziomyces* sp.-qR	CACAAGTTTACCCAGTCATTTCG
*Nl18S*-qF	GTAACCCGCTGAACCTCC
*Nl18S*-qR	GTCCGAAGACCTCACTAAATCA

## Data Availability

The original contributions presented in this study are included in the article/Supplementary Materials. Further inquiries can be directed to the corresponding author.

## Supplementary Materials

The following supporting information can be downloaded at https://www.mdpi.com/article/10.3390/insects16090895/s1, Figure S1: Validation the specificity of the PSMC4 antibody through Western blot.
