# Supplementary figures and images for: Proteasome Subunits Regulate Reproduction in Nilaparvata lugens and the Transovarial Transmission of Its Yeast-like Symbionts
Source: Insects. 2025 Aug 27;16(9):895. doi: 10.3390/insects16090895 (PMC12470604; doi:10.3390/insects16090895)

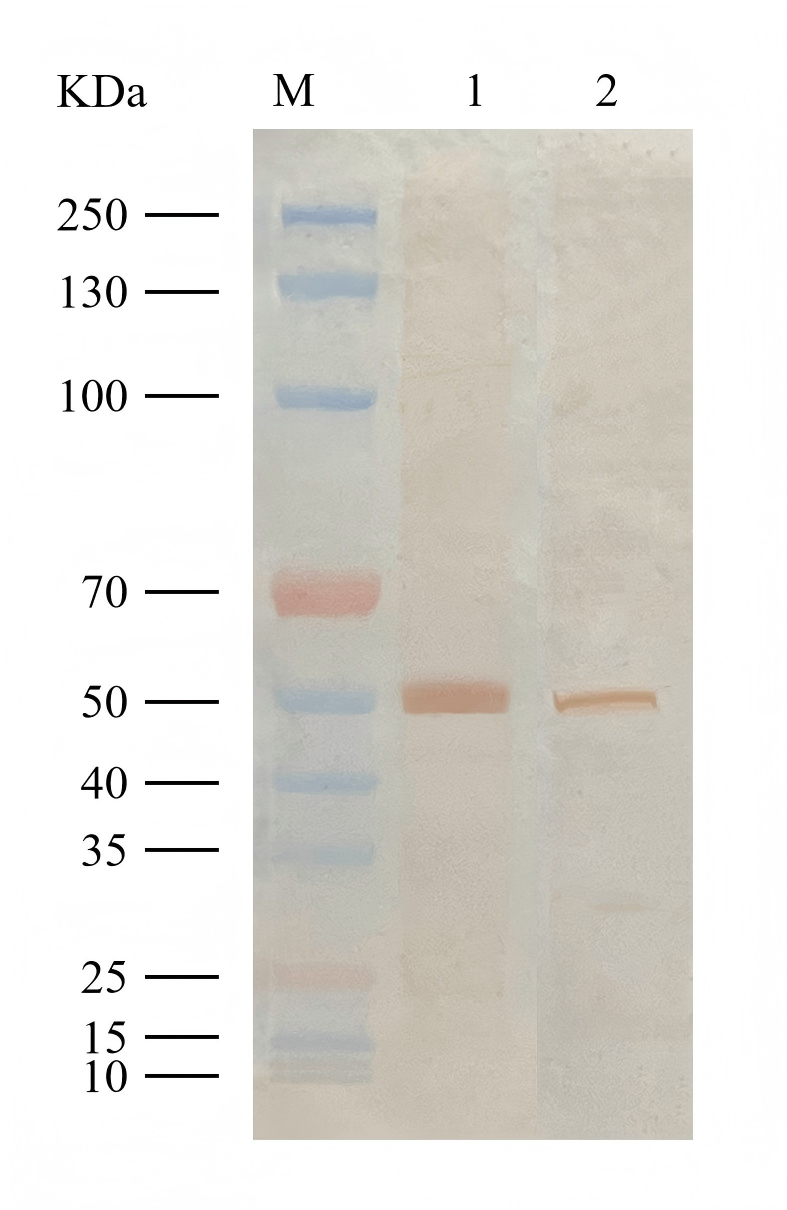

Supplement: Supplementary file 1 [file insects-16-00895-s001.zip › Figure S1.png]
